# Factors associated with physical activity following total knee arthroplasty for knee osteoarthritis: a longitudinal study

**DOI:** 10.1186/s12891-024-07306-3

**Published:** 2024-02-27

**Authors:** Remi Fujita, Susumu Ota, Yuri Yamamoto, Akito Kataoka, Hideki Warashina, Takahiro Hayashi, Naomichi Matsunaga, Hideshi Sugiura

**Affiliations:** 1 Department of Integrated Health Sciences, Nagoya University Graduate School of Medicine, Nagoya, Japan; 2 Department of Rehabilitation and Care, Seijoh University, 2-172 Fukinodai, Tokai, 476-8588 Aichi Japan; 3 Department of Rehabilitation, Nagoya Orthopaedic and Joint Replacement Clinic, Kitanagoya, Japan; 4 Department of Orthopedics, Nagoya Orthopaedic and Joint Replacement Clinic, Kitanagoya, Japan

**Keywords:** Knee osteoarthritis, Total knee arthroplasty, Physical activity

## Abstract

**Background:**

After total knee arthroplasty (TKA), patients’ physical activity (PA) levels at 6 months are lower than those of healthy subjects. Few studies have investigated the factors associated with PA at 6 months after TKA by objectively measuring preoperative and postoperative PA intensity using an accelerometer and knee function using a goniometer and dynamometer. The purpose of this study was to determine the factors associated with PA levels at 6 months after TKA based on objective data.

**Methods:**

Eighty-two patients (mean [SD] age 74.5 [6.4] years) with moderate-to-severe knee osteoarthritis (OA) who were scheduled for TKA at the Nagoya Orthopaedic and Joint Replacement Clinic from July 2018 to July 2019 were enrolled in this longitudinal study. All patients underwent evaluations of knee function, including range-of-motion and knee-extension muscle strength; knee pain; performance in the timed up-and-go test; and accelerometer-measured PA both preoperatively and 6 months postoperatively. Factors associated with PA at 6 months after TKA were assessed using a hierarchical multiple linear regression analysis adjusted for age, sex, body mass index, and presence of diabetes mellitus.

**Results:**

A higher average daily step count at 6 months after TKA was significantly associated with greater preoperative knee-extension muscle strength on the operated side (*β* = 0.155, *p* = 0.028) as well as a higher preoperative average daily step count (*β* = 0.834, *p* < 0.001). Furthermore, average daily time spent in moderate-to-vigorous-intensity PA postoperatively was significantly associated only with time spent in moderate-to-vigorous-intensity PA preoperatively (*β* = 0.723, *p* < 0.001).

**Conclusion:**

These findings indicate that a higher preoperative daily step count and greater preoperative knee-extension muscle strength on the operated side may be associated with a higher daily step count at 6 months after TKA. Factors associated with PA differed by the PA intensity level. Rehabilitation and interventions for psychosocial factors before TKA beginning when mild knee OA first occurs are expected to lead to increased PA in TKA patients.

**Supplementary Information:**

The online version contains supplementary material available at 10.1186/s12891-024-07306-3.

## Introduction

Osteoarthritis (OA) of the knee, the most commonly affected joint worldwide, is a leading cause of pain, disability, and socioeconomic loss [1, 2]. Knee OA patients often limit their daily physical activity (PA) due to severe pain [3]. Total knee arthroplasty (TKA) is considered to be an effective treatment for end-stage knee OA [4]. The aims of TKA are to restore joint function, mitigate severe knee pain, and minimize functional disability [5], thereby improving PA levels of patients with knee OA and minimizing their disability. Observational studies indicate that most of the improvement in pain, physical function, and 6-minute walk distance (a parameter of functional performance) occurs by 3 months postoperatively, with improvement plateauing by 6 months [6–8].

The amount of PA after TKA is associated with sex, body mass index (BMI), age, renal failure, neurological disorders, preoperative PA, and disease-specific quality of life [9–15]. In previous studies, PA or knee function was assessed using questionnaires and only patient characteristics were used as independent variables; although questionnaires are a simple and versatile method of assessing PA, potential uncertainty in recall and overestimation of PA may affect the accuracy of the results [16–18]. Accurately quantifying PA has become easier with advances in measurement instruments, and compared with questionnaires, measurements of PA by an accelerometer provide reliable and objective intensity-specific data [19, 20]. Measurements by accelerometers can be tracked over a continuous, longer period of time in a free-living environment, providing insight into a patient’s actual PA patterns [21]. In addition, patient characteristics and preoperative patient-reported knee function, knee pain, physical function, and quality of life alone are not considered sufficiently reliable factors associated with postoperative PA [22]. Therefore, accurate measurements of preoperative and postoperative PA levels based on the intensity as well as objective measurements of preoperative knee function and physical function are necessary to evaluate the factors associated with postoperative PA. Studies investigating factors associated with PA have analyzed patient characteristics as adjustment factors, including age, sex, BMI, and presence of diabetes mellitus (DM) [14]. Previous studies of knee OA emphasized that type 2 DM comorbidity in patients with knee OA is linked to the development [23, 24] and progression [25] of knee OA, and we therefore examined the presence of DM as an adjustment factor. Physical activity after TKA, however, reportedly remains lower than that in healthy subjects [8].

The present study had the following aims: (1) to describe the changes in PA from the preoperative period to 6 months postoperatively in patients with knee OA undergoing TKA, and (2) to determine factors associated with the step count and intensity level of PA at 6 months after TKA using objective data. Identifying the factors associated with PA after TKA will contribute to the development of appropriate and specific rehabilitation interventions.

## Methods

### Study design and setting

This study was a longitudinal study with 2 measurement points carried out at the Nagoya Orthopaedic and Joint Replacement Clinic (Kitanagoya, Japan) between July 2018 to January 2020. Data were prospectively collected from the outpatient department. The study was conducted according to the principles of the Declaration of Helsinki and approved by the Ethics Committee of Seijoh University (Tokai, Japan; approval no. 2018A0006) and Nagoya University (Nagoya, Japan; approval no. 20–521). All methods were conducted in accordance with the relevant guidelines and regulations. All patients provided written informed consent.

### Patients

**Fig. 1:**
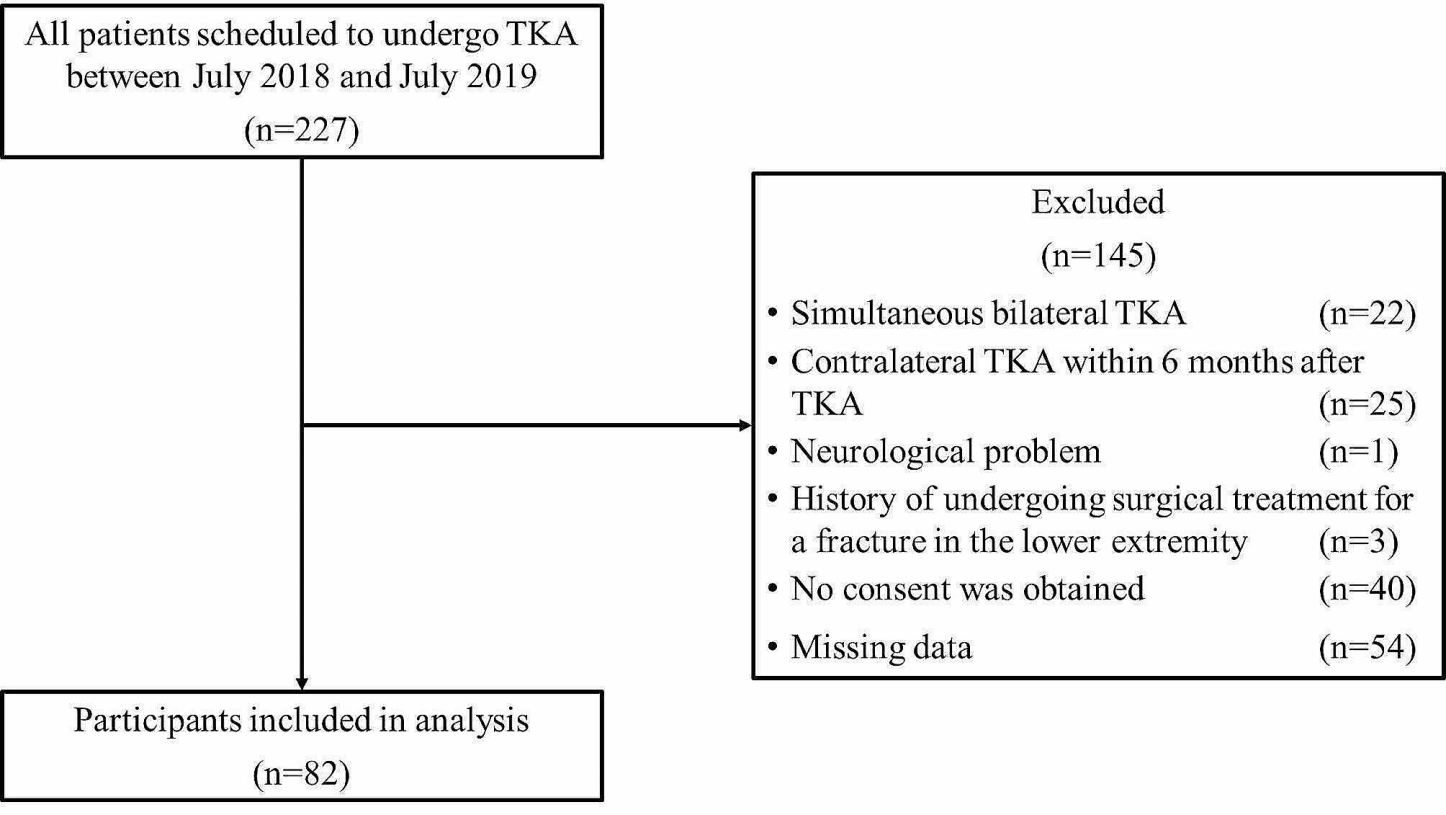
Flow diagram of the inclusion/exclusion criteria for the analysis before TKA. TKA, total knee arthroplasty

Middle- and older-aged patients (≥ 55 years) having radiographically confirmed knee OA (i.e., Kellgren‒Lawrence [KL] grade ≥ 2 in at least 1 knee [26]) who were scheduled for TKA were enrolled in the study. Patients were included if they underwent TKA from July 2018 through July 2019, and were able to walk independently on a flat surface with or without an assistive device. Exclusion criteria were as follows: (1) simultaneous bilateral TKA, (2) contralateral TKA within 6 months after TKA, (3) cognitive impairment, (4) neurological problems, (5) history of surgical treatment for lower extremity and/or spinal fracture, (6) did not provide consent, or (7) cases with missing data. The process for inclusion in the analysis is shown in Fig. 1.

### Demographic characteristics

Clinical records were used to collect data on age, sex, height, weight, and radiographic OA severity. The BMI of each patient was calculated by dividing the weight (kg) by the square of the height (m). A trained examiner (orthopedist) assessed the radiographic OA severity in the tibiofemoral joint based on KL grades [26] in the anteroposterior and weight-bearing position for each patient. DM was defined as an elevated fasting plasma glucose level ≥ 126 mg/dL, HbA1c level ≥ 6.5%, a formal diagnosis of DM, or prescribed antidiabetic medications [27].

### Measures

All clinical data were collected 1‒4 weeks before TKA and at 6 months after TKA. Outcome measures were knee function (knee flexion and extension range-of-motion [ROM], and knee-extension muscle strength), knee pain, performance-based physical function (measured with the timed up-and-go [TUG] test), and objectively measured PA.

#### Knee function

Knee flexion and extension ROM were assessed as the angle formed by the intersection of the mechanical axes of the femur (i.e., line from the greater trochanter to the lateral femoral epicondyle) and lower leg (i.e., line from the head of the fibula to the lateral malleolus of the fibula). With patients positioned supine, passive knee flexion and extension ROM were measured using a standard goniometer.

Knee-extension muscle strength during isometric contraction was assessed using an isometric dynamometer (Isoforce GT-360; OG Wellness Technologies Co., Ltd., Okayama, Japan). For the measurements, patients were seated upright on the dynamometer with the tested limb’s knee flexed at a 60° angle. The chair depth, height, placement, and length of the attachment arm were customized for each patient. To ensure stability, straps were used to secure the patient at the distal shank, mid-thigh, pelvis, and trunk. Patients were instructed to achieve maximum contraction within 3 s and to sustain the contraction for 5 s. Throughout the process, standardized verbal encouragement was given to motivate the patients to exert their maximum effort. The resulting maximum knee-extension muscle strength values were then recorded [28, 29]. Each patient underwent 2 trials and the maximum muscle strength reached during the 2 trials was normalized to the patient’s body weight. The normalized values were used for data analysis.

#### Knee pain

A 100-mm visual analog scale for pain was used to measure the pain intensity in the operated and contralateral knees during the preceding week.

#### Performance-based physical function measure

Performance-based physical function was objectively assessed according to the TUG test (i.e., ambulatory transitions) as recommended by the Osteoarthritis Research Society International. The TUG test [30] is a simple, commonly used, and reliable test for clinical application in patients with suspected knee OA [31, 32]. Patients were directed to transition from a seated position with a seat height of 40 cm to standing. They were then instructed to walk a distance of 3 m at a normal pace, return to the starting point, and resume a seated position unaided (although assistive devices were permitted if needed). The time required to perform this test once was measured using a stopwatch.

#### Physical activity (PA)

A uniaxial accelerometer (Lifecorder; Suzuken Co., Ltd., Nagoya, Japan) was used to measure PA for 14 consecutive days. The reliability, validity, consistency, and accuracy of the Lifecorder accelerometer for estimating the step count and intensity of walking activity has been established under both controlled and free-living conditions [33, 34], and it is widely used in studies of PA [35, 36]. Patients were given both oral and written explanations regarding the proper use of the accelerometer. The accelerometer was attached at waist level on the midline of the right or left thigh and worn throughout the day. It was to be removed only for sleep and water-related activities, such as bathing, showering, and swimming. For inclusion in the study, patients had to wear the accelerometer for ≥ 10 h/d for ≥ 4 days (including 1 weekend day) [37]. “Nonwear time” was defined as a period of at least 60 consecutive minutes when no body movement data were recorded, except for up to 2 min of limited movement [37]. Of the 14 days, all days that met the inclusion criteria were used in the analysis. The accelerometer data were edited and aggregated using specialized PA analysis software (LifeLyzer05 Coach; Suzuken Co., Ltd., Nagoya, Japan). Based on the 11 exercise intensity levels from the accelerometer, which records signals at intensity levels of 0, 0.5, or 1–9 every 4 s while being worn), PA intensity levels were categorized as light (levels 1–3; ≥1.5 to < 3 metabolic equivalents [METs]), moderate (levels 4–6; ≥3 to < 6 METs), or vigorous (levels 7–9; ≥6 METs) [35]. The average daily step count (measured as steps per day) and duration of PA at each respective intensity level (measured as time in minutes per day) were calculated. For the majority of the patients (~ 70%), no time was spent in vigorous-intensity PA; therefore, a single variable was constructed combining the time spent in moderate-intensity PA with the time spent in vigorous-intensity PA [38]. In older people, the majority of daily step counts reflects light intensity (< 3 METs) activity [39]. The overall health of older people is associated with both the year-averaged daily duration of moderate-to-vigorous-intensity PA (MVPA) and the year-averaged daily step count [40]; therefore, final variables were determined as daily step count and time spent in MVPA.

### Data analyses

Statistical analyses were performed using IBM SPSS Statistics for Windows, version 25 (IBM Corp., Armonk, NY, USA). Descriptive statistics were calculated as the mean ± standard deviation for continuous variables and as the proportion for dichotomous/categorical variables. The normality of the continuous variables was determined using the Shapiro‒Wilk test. The Student’s *t*-test for parametric continuous variables and the Wilcoxon signed-rank test for nonparametric continuous variables were used to compare PA before TKA and at 6 months after TKA.

To assess the factors influencing PA at 6 months after TKA, a hierarchical multiple linear regression analysis was performed. The PA parameters, including the average daily step count and time spent in MVPA were utilized as dependent variables. Age, sex, BMI, and the presence of DM (0 = without DM, 1 = with DM) prior to TKA were included as covariates for the analysis. These adjustment variables were selected a priori based on clinical judgment due to their potential influence on PA; that is, older people, women, individuals with higher BMI, and individuals with DM have lower levels of PA [14, 41]. Correlations between PA parameters at 6 months after TKA and knee flexion and extension ROM, knee-extension muscle strength, knee pain, and TUG before TKA were analyzed using Pearson’s correlation coefficient when the variables were normally distributed and Spearman’s rank correlation coefficient when the variables were not normally distributed. Variables determined to be significant (*p* < 0.05) in the correlation analysis were included as independent variables in a hierarchical multiple linear regression analysis to evaluate factors associated with PA parameters at 6 months after TKA [42, 43]. Each model had 2 steps. In step 1, the adjustment variables were included using the forced-entry method. In step 2, the independent variables were further included using the stepwise method. All independent variables were screened for multicollinearity by calculating the variance inflation factor. The independence of residual errors was assessed using the Durbin‒Watson statistic, which revealed independence in the range of 1.5‒2.5. The standardized partial regression coefficient (*β*) was used to interpret the significance of correlations. A *p* value < 0.05 was considered statistically significant.

Post hoc power analysis was conducted to verify the sample size using G*Power [44]. The post hoc power of a total sample size of 82 and 7 independent variables was 98% and 67% for effect sizes assumed to be large and medium, respectively. The number of subjects used in this study was therefore sufficiently large to detect statistically significant differences.

## Results

The preoperative characteristics of the 82 patients included in the analysis are summarized in Table 1. The mean (standard deviation) patient age was 74.5 (6.4) years, and 84.1% were women.

**Table 1:** Summary of the preoperative characteristics of participants (*n* = 82)

Variable	Mean ± SD or n (%)
Demographic characteristic	
Age (y)	74.5 ± 6.4
Sex	
Men	13 (15.9)
Women	69 (84.1)
Height (cm)	153.9 ± 7.7
Weight (kg)	60.1 ± 11.7
BMI (kg/m^2^)	25.2 ± 3.7
Diabetes mellitus	
Yes	22 (26.8)
No	60 (73.2)
Severity of knee OA	
Kellgren‒Lawrence grade on operated side (3/4)	15/ 67 (18.3/ 81.7)
Kellgren‒Lawrence grade on contralateral side (2/3/4/operated/unknown)	11/ 11/ 31/ 27/ 2 (13.4/ 13.4/ 37.8/ 32.9/ 2.4)
Knee function	
Knee-flexion ROM (°)	
Operated side	130.0 ± 12.8
Contralateral side	131.6 ± 12.6
Knee-extension ROM (°)	
Operated side	-5.9 ± 5.1
Contralateral side	-3.5 ± 5.1
Knee-extension muscle strength (Nm/kg)	
Operated side	1.02 ± 0.34
Contralateral side	1.14 ± 0.37
Knee pain, VAS (mm)	
Operated side	51.3 ± 24.0
Contralateral side	27.1 ± 24.0
Performance-based physical function measure	
TUG test time (s)	11.9 ± 4.9

BMI, body mass index; OA, osteoarthritis; ROM, range-of-motion; VAS, visual analog scale; TUG, timed up-and-go; SD, standard deviation

### Changes in PA from the preoperative period to 6 months postoperatively

Both the average daily step count and MVPA time were significantly increased at 6 months after TKA compared with preoperative levels (Table 2).

**Table 2:** Physical activity assessed preoperatively and 6 months postoperatively

Assessments	Preoperative	6 months postoperative	p-value	Effect size
Average daily step count (steps/day)	4717.7 ± 2533.1	5353.8 ± 2706.9	**< 0.001**	0.433
Time spent in MVPA (min/day)	4.5 ± 5.6	7.9 ± 7.9	**< 0.001**	0.665

MVPA, moderate-to-vigorous-intensity physical activity

Physical activity was analyzed using the Wilcoxon signed-rank test

Note: Data are expressed as means ± standard deviations. Statistically significant p values (*p* < 0.05) are shown in bold font. Effect size: <0.1 = trivial effect, 0.1–0.3 = small effect, 0.3–0.5 = medium effect; and > 0.5 = large effect

### Correlations between PA parameters at 6 months after TKA and the variables before TKA

Correlation analysis between PA at 6 months after TKA and knee function, knee pain, the TUG test time, and PA before TKA revealed that PA at 6 months after TKA was significantly correlated with several parameters (Table 3).

**Table 3:** Spearman correlation coefficients for associations between physical activity 6 months postoperatively and variables measured preoperatively

	Average daily step count 6 months postoperatively		Time spent in MVPA 6 months postoperatively	
Variables	Correlation coefficient	p-value	Correlation coefficient	p-value
Knee-flexion ROM operated side preoperatively	**0.238**	**0.020**	**0.214**	**0.037**
Knee-flexion ROM contralateral side preoperatively	**0.286**	**0.005**	**0.245**	**0.016**
Knee-extension ROM operated side preoperatively	0.135	0.193	0.099	0.340
Knee-extension ROM contralateral side preoperatively	**0.210**	**0.041**	0.170	0.099
Knee-extension muscle strength operated side preoperatively	**0.312**	**0.002**	**0.295**	**0.004**
Knee-extension muscle strength contralateral side preoperatively	**0.323**	**0.001**	**0.356**	**< 0.001**
Knee pain, VAS operated side preoperatively	-0.036	0.728	-0.144	0.164
Knee pain, VAS contralateral side preoperatively	0.071	0.494	-0.090	0.384
TUG test time preoperatively	**-0.344**	**0.001**	**-0.396**	**< 0.001**
Average daily step count preoperatively	**0.845**	**< 0.001**	–	–
Time spent in MVPA preoperatively	–	–	**0.763**	**< 0.001**

ROM, range-of-motion; VAS, visual analog scale; TUG, timed up-and-go; MVPA, moderate-to-vigorous-intensity physical activity

Note: Statistically significant p values (*p* < 0.05) are shown in bold font

### Factors associated with the step count and MVPA at 6 months after TKA

A hierarchical multiple linear regression analysis, after adjusting for age, sex, BMI, and presence of DM, revealed that a higher average daily step count at 6 months after TKA was significantly associated with greater preoperative knee-extension muscle strength on the operated side (*β* = 0.155; *p* = 0.028) and a higher preoperative average daily step count (*β* = 0.834; *p* < 0.001) (Supplementary Table 1). In addition, a longer MVPA time at 6 months after TKA was significantly associated with a longer preoperative MVPA time (*β* = 0.723; *p* < 0.001; Supplementary Table 1).

The variance inflation factor was < 10, indicating that no collinearity existed among the variables and that none of the significant relationships were inflated by correlations between independent variables [45]. Residual plots indicated a random distribution pattern. Independence was observed among the residual errors of average daily step count and time spent in MVPA at 6 months after TKA (Durbin–Watson statistic: 2.059 and 2.195, respectively). These findings confirmed the validity of the multivariate regression analysis results.

## Discussion

The findings of the present study indicate that the average daily step count and MVPA time were significantly increased at 6 months after TKA compared with preoperative levels. These results are consistent with those of a previous systematic review [8]. In the present study, we only used an accelerometer to objectively measure PA, in contrast to the previous study, which included both accelerometer and self-report data. The divergence between self-report and accelerometer-based measures is unknown. Nevertheless, the consistent results between our study and those of the previous study suggest the accuracy of the PA measurements.

In addition, our results indicated that the greater the preoperative knee-extension muscle strength on the operated side and the higher the preoperative average daily step count, the higher the average daily step count at 6 months after TKA. Further, the longer the preoperative MVPA time, the longer the MVPA time at 6 months after TKA. Preoperative knee-extension muscle strength is a predictor of abnormal movement patterns during gait at 6 months after TKA [46]. Consistent with this previous finding, the present study demonstrated that preoperative knee-extension muscle strength on the operated side was associated with the average daily step count at 6 months after TKA. The effect size of the preoperative knee-extension muscle strength on the operated side, however, was smaller (*β* = 0.155) than that of the preoperative daily step count on the daily step count 6 months after TKA (*β* = 0.834). Some consistent correlates of PA are individual-level factors such as age, sex, health status, intention to exercise, self-efficacy, and previous PA, in addition to factors related to walking ability [41]. Physical environmental factors (e.g., transport, places to exercise, leisure, occupation, and home) as well as social environmental factors (e.g., economic conditions, societal norms, urbanization, and industrialization), are associated with PA [41]. In addition, genetic factors contributing to the propensity to be physically active, such as likes and dislikes of exercise, craving activity, feeling rewarded by accomplishing an activity, and experiencing fatigue, are associated with PA [41]. Gait speed after TKA is reported to be associated with the amount of change postoperatively in knee-flexion ROM and knee-extension muscle strength, with knee-extension muscle strength on the operated side showing the strongest association [47]. We expect that psychological, environmental, and genetic factors, and postoperative knee-extension muscle strength on the operated side, which were not measured in this study, influenced preoperative knee-extension muscle strength on the operated side, leading to a small standardized partial regression coefficient (*β*).

Daily step count and MVPA time at 6 months after TKA were significantly associated with the preoperative daily step count and MVPA time, respectively. Our findings are consistent with those of other studies reporting the influence of preoperative PA on postoperative PA [9, 12], and confirm that the level of preoperative PA is an important factor affecting the level of postoperative PA.

Exercise therapy, including muscle strengthening, aerobic exercise, and balance training, is the primary recommended nonsurgical treatment for knee OA. Additional recommendations for nonsurgical treatment of knee OA include the use of walking aids and patient education regarding a healthy lifestyle, such as regular PA [48, 49]. In addition, current guidelines recommend preoperative exercise therapy to increase muscle strength and flexibility and daily PA, including moderate- to high-intensity aerobic exercise on land and in the water, although the number of high-quality studies on which these guidelines are based is limited [50].

Multilevel interventions that alter both psychosocial and environmental variables may be most effective in increasing PA [51]. In areas with walkable communities, individually targeted behavior change techniques may be especially beneficial [51]. Therefore, although it is difficult to provide support for environmental factors such as access to places where people can exercise, it is easy to provide support for increasing walking ability and psychological factors. In the present study, knee-extension muscle strength before TKA was positively and significantly correlated with the daily step count after TKA, suggesting that strengthening knee-extension muscles before TKA may be effective for increasing PA after TKA. Based on previous studies [41, 48–51] and the results of this study, a behavior modification approach should be initiated when knee OA is mild.

Preoperative knee-flexion ROM on the operated side, knee-flexion ROM on the contralateral side, knee-extension ROM on the contralateral side, knee-extension muscle strength on the contralateral side, and the TUG test time, which were significantly correlated with the daily step count and MVPA time 6 months after TKA in the single correlation analysis, were not extracted as relevant factors in the hierarchical multiple linear regression analysis.

Knee-extension muscle strength on the contralateral side and the TUG test time had correlation coefficients between approximately ± 0.5 and ± 0.7 in the single correlation analysis with knee-extension muscle strength on the operated side, showing the highest correlation in the single correlation analysis among the independent variables measured in this study (*r* = 0.656, -0.474, respectively). The TUG test time also showed a high correlation with age (*r* = 0.512). This may have led to the apparent association in the single correlation analysis.

In a previous study on the relationship between knee-extension ROM, knee-extension muscle strength, and the physical function score on the 36-item short-form survey (SF-36 physical function), the amount of change in knee-extension muscle strength (before and after TKA) mediated the association between the amount of change in knee-extension ROM and SF-36 physical function [52]. Gait speed after TKA was also associated with the amount of postoperative change in knee-flexion ROM and in knee-extension muscle strength, with knee-extension muscle strength on the operated side showing the strongest association [47]. In these reports, PA was assessed on the basis of a questionnaire and gait speed, but the results may explain why the association between knee ROM and PA in the single correlation analysis no longer held in the multiple regression analysis.

For knee pain, neither the daily step count nor the MVPA time showed significant correlations in the single correlation analysis. A previous study reported that gait speed after TKA was not associated with knee pain before TKA [47]. Further, PA was not associated with knee pain in patients with knee OA [53], and severe knee pain 2 weeks after TKA was associated with shorter daily walking time [54]. Therefore, because knee pain early after TKA may be more strongly associated with the amount of PA than knee pain before TKA, there was no association of knee pain before TKA with the amount of PA 6 months after TKA.

Another study reported that factors associated with PA after TKA based on responses to a questionnaire included sex, preoperative PA, renal failure, and neurological disorders [9, 14]. Factors associated with PA after TKA measured using an accelerometer were sex, BMI, and self-reported knee function [10, 11]. In the present study, we extracted the knee-extension muscle strength on the operated side from knee function as a relevant factor and objective evaluation of all items indicated the reliability of the results.

A strength of this study is the analysis of factors associated with postoperative PA based on objective measurements of preoperative and postoperative PA, preoperative knee function, and physical function. Most previous studies investigated these factors using questionnaires. We believe that our study is the first to investigate the factors associated with postoperative PA using objectively measured indices. Nevertheless, this study has several limitations that must also be considered. First, 54 patients (12 men, 42 women [mean age 75.2 ± 7.4 years, mean BMI 24.7 ± 3.0 kg/m^2^], 12 with DM, 42 without DM, 9 KL grade 3, and 45 KL grade 4 on the operated side) were excluded due to missing data. There were no significant differences in any of the preoperative characteristics between the final study participants and the excluded patients. Therefore, the effect of exclusion of patients with missing data on the results is considered to be small. Second, this study focused on PA at 6 months after TKA because improvement in pain, physical function, and functional performance is reported to reach a near plateau at 6 months postoperatively [6–8]. The long-term course of PA beyond 6 months postoperatively, however, has been reported [8], and PA not only at 6 months postoperatively but also after 6 months postoperatively is important for patients with TKA. Therefore, future studies of the long-term course of PA after TKA should be performed with the same methodology used in the present study. Third, certain types of PA (e.g., water-related activities and cycling) or posture cannot be tracked by accelerometers. Fourth, factors other than age, sex, and physical factors that correlate with PA, such as psychological, psychosocial, and environmental factors [40, 41, 55], were not investigated in this study.

## Conclusion

Greater preoperative knee-extension muscle strength on the operated side as well as a higher preoperative daily step count may be associated with a higher daily step count at 6 months after TKA. Factors associated with PA differed by PA intensity level, but the preoperative daily step count was more strongly associated with the daily step count at 6 months after TKA than with preoperative knee-extension muscle strength before TKA.

Based on previous studies as well as the results of this study, rehabilitation focused on strengthening knee-extension muscle strength on the operated side and interventions for psychosocial factors before TKA and beginning when knee OA is mild might be effective for increasing PA in TKA patients. A prospective intervention study is needed to confirm this in the future.

### Electronic supplementary material

Below is the link to the electronic supplementary material.

Supplementary Material 1

## Data Availability

The datasets used and analyzed in the present study are available from the corresponding author upon reasonable request.

## Abbreviations

BMI: Body mass index
DM: Diabetes mellitus
KL: Kellgren‒Lawrence
METs: Metabolic equivalents
MVPA: Moderate-to-vigorous-intensity physical activity
OA: Osteoarthritis
PA: Physical activity
ROM: Range-of-motion
SD: Standard deviation
TKA: Total knee arthroplasty
TUG: Timed up-and-go
