# Supplementary material for: Factors associated with physical activity following total knee arthroplasty for knee osteoarthritis: a longitudinal study
Source: BMC Musculoskelet Disord. 2024 Feb 27;25:178. doi: 10.1186/s12891-024-07306-3 (PMC10898134; doi:10.1186/s12891-024-07306-3)
Supplement: Supplementary file 1 — Supplementary Material 1 [file 12891_2024_7306_MOESM1_ESM.docx]

**Additional file 1**

**Supplementary Table 1** Hierarchical multiple regression analysis of factors associated with physical activity 6 months postoperatively

| Variables | Step 1 |  |  |  | Step 2 |  |  |
| --- | --- | --- | --- | --- | --- | --- | --- |
|  | *B* | *β* | p-value |  | *B* | *β* | p-value |
| **Average daily step count** |  |  |  |  |  |  |  |
| Intercept | 25206.5 |  | < 0.001 |  | 380.5 |  | 0.917 |
| Age | -210.6 | -0.511 | < 0.001 |  | 4.2 | 0.010 | 0.898 |
| Sex (0: men; 1: women) | 801.6 | 0.111 | 0.268 |  | 869.2 | 0.121 | 0.072 |
| BMI | -182.6 | -0.255 | 0.015 |  | -63.3 | -0.088 | 0.197 |
| DM (0: without DM; 1: with DM) | -1205.8 | -0.203 | 0.049 |  | 202.2 | 0.034 | 0.610 |
| Knee-extension muscle strength  operated side preoperatively |  |  |  |  | 1218.2 | 0.155 | **0.028** |
| Average daily step count  preoperatively |  |  |  |  | 0.9 | 0.834 | **< 0.001** |
| Adjusted *R*^2^ | 0.317 | | |  | 0.739 | | |
| **Time spent in MVPA** |  |  |  |  |  |  |  |
| Intercept | 3503.0 |  | < 0.001 |  | 898.7 |  | 0.136 |
| Age | -32.8 | -0.459 | < 0.001 |  | -5.9 | -0.082 | 0.353 |
| Sex (0: men; 1: women) | 78.2 | 0.063 | 0.561 |  | 89.5 | 0.072 | 0.357 |
| BMI | -24.8 | -0.199 | 0.074 |  | -13.3 | -0.107 | 0.187 |
| DM (0: without DM; 1: with DM) | -149.6 | -0.145 | 0.188 |  | -25.5 | -0.025 | 0.759 |
| Time spent in MVPA  preoperatively |  |  |  |  | 1.0 | 0.723 | **< 0.001** |
| Adjusted *R*^2^ | 0.209 | | |  | 0.589 | | |

BMI, body mass index; DM, diabetes mellitus; MVPA, moderate-to-vigorous-intensity physical activity

*Note: B* is the partial regression coefficient and *β* is the standardized partial regression coefficient. Statistically significant p values (p<0.05) are shown in bold font
